# The coding and noncoding transcriptome of *Neurospora crassa*

**DOI:** 10.1186/s12864-017-4360-8

**Published:** 2017-12-19

**Authors:** Ibrahim Avi Cemel, Nati Ha, Geza Schermann, Shusuke Yonekawa, Michael Brunner

**Affiliations:** 1 Heidelberg University Biochemistry Center, 69120 Heidelberg, Germany; 2 present address: Cellzome GmbH, 69117 Heidelberg, Germany; 3 present address: Yoshida & Co., Ltd., Tokyo, 151-8580 Japan

**Keywords:** *Neurospora*, Transcriptome, Noncoding RNA, Antisense, Splicing

## Abstract

**Background:**

Long non protein coding RNAs (lncRNAs) have been identified in many different organisms and cell types. Emerging examples emphasize the biological importance of these RNA species but their regulation and functions remain poorly understood. In the filamentous fungus *Neurospora crassa,* the annotation and characterization of lncRNAs is incomplete.

**Results:**

We have performed a comprehensive transcriptome analysis of *Neurospora crassa* by using ChIP-seq, RNA-seq and polysome fractionation datasets*.* We have annotated and characterized 1478 long intergenic noncoding RNAs (lincRNAs) and 1056 natural antisense transcripts, indicating that 20% of the RNA Polymerase II transcripts of *Neurospora* are not coding for protein. Both classes of lncRNAs accumulate at lower levels than protein-coding mRNAs and they are considerably shorter. Our analysis showed that the vast majority of lincRNAs and antisense transcripts do not contain introns and carry less H3K4me2 modifications than similarly expressed protein coding genes. In contrast, H3K27me3 modifications inversely correlate with transcription of protein coding and lincRNA genes. We show furthermore most lincRNA sequences evolve rapidly, even between phylogenetically close species.

**Conclusions:**

Our transcriptome analyses revealed distinct features of *Neurospora* lincRNAs and antisense transcripts in comparison to mRNAs and showed that the prevalence of noncoding transcripts in this organism is higher than previously anticipated. The study provides a broad repertoire and a resource for further studies of lncRNAs.

**Electronic supplementary material:**

The online version of this article (10.1186/s12864-017-4360-8) contains supplementary material, which is available to authorized users.

## Background

Recent genome-wide transcriptome analyses have documented the complexity of eukaryotic transcriptomes [1–3] and revealed that a substantial portion of the genome gives rise to non-protein coding RNAs (ncRNAs) [4, 5]. ncRNA species share common post-transcriptional modifications with protein-coding messenger-RNAs (mRNAs), including splicing and polyadenylation signals [6] but lack an open reading frame. These RNA types include ribosomal RNAs, transfer RNAs, microRNAs and long non-coding RNAs (lncRANs). In the past decade, particular attention was given to the ever-expanding class of lncRNAs, which are predicted to regulate important cellular processes, such as dosage compensation, imprinting, regulation of chromatin states [7–10], cell fate determination [11] and gene silencing [12, 13]. Nonetheless, the function of most lncRNAs remains uncharacterized.

One class of lncRNAs is natural antisense transcripts, which are transcribed from overlapping loci on the opposite strand of the DNA. They were found to regulate gene expression and thus provide an additional level of control by RNA-mediated mechanisms [14, 15]. Antisense RNAs may or may not code for proteins. They have been reported to exert their regulatory effects on the corresponding sense mRNAs via epigenetic regulation, chromatin remodeling [16–18], RNA-RNA interactions and post-transcriptional mechanisms, including regulation of mRNA processing and transport [19, 20]. The prevalence of lncRNAs has been reported in a wide range of eukaryotic organisms including plants [21], fungi [22, 23] and mammals [6, 14].

The 40-megabase genome of *Neurospora crassa* harbors 9730 protein-coding genes [24] and a broad spectrum of long intergenic ncRNAs (lincRNAs) as well as antisense transcripts [23]. In *Neurospora*, a well-documented example of an antisense lncRNA, *qrf,* was shown to regulate the core circadian clock gene *frequency (frq)* [25, 26]. Furthermore, the antisense transcript was found to be necessary to establish DNA methylation at the *frq* promoter [27].

Here we annotate and characterize 1478 lincRNAs and 1056 antisense transcripts in *Neurospora crassa* using published and new deep-sequencing data that includes RNA-seq, RNA polymerase II (RNAPII) ChIP-seq and polysome fractionation. Our data indicate that about 20% of the RNA polymerase II (RNAPII) transcripts of *Neurospora* are non-coding. We also confirmed annotated splice sites and identified new splice junctions and alternative splice sites, which are rare in *Neurospora*. In addition, we provide for the scientific community to access our webpage, which features a collection of genome-wide deep sequencing data for the model *Neurospora crassa*.

## Methods

### Unit detection pipeline and lincRNA detection

The *N. crassa* genome (NC10 genome model) was segmented into non-overlapping 50 base pair (bp) units (bins). αN-terminus RNAPII ChIP-seq [28] and pooled RNA-seq datasets [29–31] (accession numbers: SRX547956, SRX547981, SRR341283.4, SRR341284.2, SRR341429.4, SRR1578070, SRR1578069, SRR1636058; total RNA-seq read count of the pooled dataset is 174,462,581) were used to quantify the mapped reads per 50 bp unit. In order to detect continuous transcription units, logistic regression model (*glm* function, R [32]) was applied:

$$ logit\left({\pi}_i\right)=\log \left(\frac{\pi_i}{1-{\pi}_i}\right)={\beta}_0+{\beta}_1{x}_{i(rna)}+{\beta}_2{x}_{i(Pol)}+e $$

In the formula, *π*_*i*_ denotes the coding indication, which assume to follow a binomial distribution (*Binomial*(*n*_*i*_, *π*_*i*_) ), while *x*_*i*(*rna*)_ and *x*_*i*(*Pol*)_ denotes the *i*_*th*_ unit coverage from RNA-seq and RNAPII ChIP-seq datasets, respectively. The training set was created by selecting 60% of the genome. Natural logarithm (ln) ratio of 1.5 was used as the cut-off value and continuous units with read counts higher than the cut-off were merged. The detected transcription units were compared to the NC10 genome model using *bedtools* [33]*.* The read counts were normalized to the length of the transcription units. The transcription units that overlapped with the annotated *Neurospora* genes were classified as coding genes and intergenic (at least 500 bp distant from a coding gene) non-overlapping transcription units were classified as possible lincRNA genes. The median read count of the polysomal RNA-seq of the detected coding regions was used to exclude intergenic transcripts present in polysomes (*n* = 434) (see text). *Wt* H3K4me2 ChIP-seq (accession number SRX550077) and *wt* H3K27me3 ChIP-seq datasets (accession number SRX1818756), which were used to characterize the presence of these histone marks on the novel intergenic transcripts (see Fig. 2a; Additional files 1 and 2: Figure S1d, e and S2b), were previously reported. The optimal averaging window size for the smoothening of the RNAPII ChIP-seq index analysis in Fig. 2a was determined based on the curve of the sum of absolute differences as the function of the window sizes.

### Strand-specific RNA-seq and antisense RNA detection

The wild type *Neurospora* strain (FGSC#2489) used in this study was acquired from FGSC. The strain was grown in standard liquid growth medium that contains 2% glucose, 0.5% L-arginine, 1× Vogel’s medium and was cultured into mats. Mycelial discs were cut out from the mats, grown for 1 day in light under constant shaking at 115 rpm at 25 °C and transferred to darkness for 24 h before light exposure (100 μE). The discs were harvested at indicated time points (0, 30, 60, 120 min). Total RNA was prepared with peqGOLD TriFAST (peqLab, Erlangen, Germany). Sequencing libraries were prepared using NEBNext Ultra Directional RNA prep Kit for Illumina (E7420). RNAs were selected by purifying polyA+ transcripts. Total read counts are 12,294,480; 20,401,017; 24,757,383 and 12,114,415 for 0, 30, 60 and 120 min time points, respectively.

The raw reads were mapped to the *Neurospora* genome (NC10) using Bowtie 2.1.0 [34]. The parameters for mapping were set to allow up to three mismatches. The counts were normalized between samples by total count and to the length of the transcription units. Similarly to lincRNA detection analysis, the *N. crassa* genome (NC10 genome model) was segmented into non-overlapping 50 bp units (bins) and logistic regression (*glm,* R [32]) was fitted for Watson and Crick strands separately (see the above given formula). The antisense transcripts were identified from the fitted model using 1.5 ln-ratio as cut-off and having minimum 10 reads per 50 bp unit. Using *bedtools* [33], overlapping sense / antisense pairs (only if both sense and antisense units contained above cut-off read counts) were selected and the identified antisense fragments were merged if they were mapped to the same annotated protein-coding gene. The pairs were further filtered based on gene distance (distance < 500 bp), in order to eliminate false-positive hits due to overlapping protein-coding genes.

### Detection of the coding genes which expressed an antisense RNA only

Sense and antisense read counts for all *Neurospora* protein coding genes were calculated with HTSeq [35] in the strand-specific RNA-seq datasets (wild type, in dark and in 30 min light). The counts were normalized between samples by total count. Loci with only antisense coverage were identified by selecting the genes, where the average sense coverage was below 1 (less than 1 sense read for every 50 bp bin) and the average antisense coverage was above 1 (more than 1 antisense read for every 50 bp bin), in both datasets. The overlapping protein-coding genes were filtered out from the outcome.

### Polyribosome (polysome) fractionation analysis

The wild type *Neurospora* strain (FGSC#2489) was used for polysome fractionation. The cultures were grown in standard liquid growth medium (2% glucose, 0.5% L-arginine, 1× Vogel’s medium) in light (100 μE) for 1 day and released to constant darkness for 22 h and were harvested at 2 h intervals. The samples were pulverized in liquid nitrogen and approximately 0.25 g of the ground mycelia was used for each experiment, the powder was resuspended in 750 μl ice-cold polysome extraction buffer (20 mM TRIS-HCl, pH 8.0, 140 mM KCl, 10 mM MgCl_2_, 1% TritonX-100, 100 μg/ml cycloheximide, 0.5% DTT and 1 mM ribonuclease inhibitor). Cycloheximide was added to the cultures prior to harvesting. Gradients containing 10-50-60% sucrose were spun for 3.5 h at 38,000 x *g* at 4 °C in an ultracentrifuge tube (Beckman ID 331374). The fractions were collected from the top of the gradient and total RNA was prepared with peqGOLD TriFAST (peqLab, Erlangen, Germany). The raw reads were mapped to the *Neurospora* genome (NC10) using Bowtie 2.1.0 [34]. The counts were normalized between samples by total count and to the length of the transcription unit. For the bioinformatics analyses, all time points were pooled in order to obtain a deeper sequencing coverage. The read count of the pooled dataset is 174,777,343.

### Splice junction detection

The splice junctions were predicted using TopHat2 (version 2.0.13 [36]) with the pooled RNA-seq datasets [29–31] (accession numbers: SRX547956, SRX547981, SRR341283.4, SRR341284.2, SRR341429.4, SRR1578070, SRR1578069, SRR1636058; total RNA-seq read count of the pooled dataset is 174,462,581). The reported intron lengths were set to minimum 50 bp and maximum 500,000 bp. The detected junctions were compared to the NC10 genome model and were divided into annotated and non-annotated junctions. The non-annotated junctions were filtered by a cut-off, defined as the quantile 25% coverage of the annotated introns. The junctions that match exactly to the annotated intron were classified as perfect matches, the junctions that varied from the annotated junction coordinates were classified as weak matches, junction pairs with shared donor or acceptor sites were classified as alternative splice isoforms and the remaining junctions were classified as novel junctions and were further analyzed (see text).

In order to detect the splice junctions in antisense transcripts, strand-specific RNA-seq datasets (wild type, in dark and in 30 min light) were mapped to *Neurospora crassa* genome with TopHat2 (version 2.0.14 [36]). 8502 splice sites with at least 10 spliced reads in either datasets were selected for the analysis (7883 had 10 or more spliced reads in both datasets). From this list of 8502 splicing sites 30 were found within the 1056 antisense RNA fragments identified (1224 sites were found within the corresponding 1056 sense transcript boundaries.)

### NEUTRA setup

The normalized RNA-seq and ChIP-seq datasets for the selected genes were plotted with Charts Google [37]. The genome browser was set up by using JBrowse [38] based on the NC10 genome model and the Wiggle files were uploaded. Next-generation sequencing data are displayed using BigWig format while the binding sites are displayed using BED format.

### Conversion of the data to NC12

The coordinates of the identified lincRNA genes, possibly coding genes, antisense transcripts and splicing junctions (Additional files 3, 4, 5, 6 and 7: Table S1,S2,S3,S4,S5) are provided in NC10 and the recently released NC12 version of the *Neurospora crassa* genome (http://fungi.ensembl.org/Neurospora_crassa) [39]. The difference between NC10 and NC12 is not substantial. Sequences of chromosomes 1 to 5 are identical. The NC12 version of chromosome 6 has the sequence from position 1,847,027 to position 2,788,223 reverse complemented while chromosome 7 has the whole sequence reverse complemented. NC10 coordinates were converted to NC12 coordinates by a custom R script [32], based on BLAT alignments [40] between the corresponding chromosomes.

## Results and discussion

### *Neurospora* transcriptome analysis

To catalogue distinct species of RNAs, such as mRNAs, lincRNAs and antisense RNAs; to investigate the structure of the annotated and novel genes in terms of their 5′ starts and 3′ ends, direction and splicing pattern; and to quantify the expression levels of the genes, we took advantage of various sequencing data. These data include published RNA-seq data from circadian, light induction and differential expression analyses of wild type (*wt*), Δ*sub1*, Δ*ff7* and Δ*csp1* [29–31] strains and an unpublished strand-specific RNA-seq. ChIP-seq analyses of RNAPII [28] and a polyribosome (polysome) fractionation analysis were also included for the *Neurospora* transcriptome analysis.

The *Neurospora crassa* genome harbors 9730 annotated protein-coding genes. By using the above mentioned RNA-seq, ChIP-seq and polysome studies, our unit detection pipeline detected 92% of the annotated genes (8956 genes), validating our methodology. The remaining 774 annotated genes were not expressed under our conditions.

### Long intergenic non-coding (linc) RNAs

To confidently determine the *Neurospora* repertoire of lincRNAs we pooled our available RNA-seq data, representing the polyadenylated transcriptome. In addition, we included an RNAPII ChIP-seq analysis [28] to confirm the boundaries of transcription units. Furthermore, to assess the non-protein-coding nature of transcripts we used RNA-seq analysis of a polysome fractionation. Non-annotated transcripts detected in the polysome fraction above the median read count of annotated coding genes (*n* = 434) were initially excluded as lincRNAs and analyzed in more detail (see below). Finally, considering that 95% of the *Neurospora* genes are more than 134 bp apart (Additional file 1: Figure S1a), and that the average intron length of *Neurospora* protein-coding genes comprises 69 bp (Additional file 1: Figure S1b), lincRNA segments that were less than 100 bp apart were merged.

**Fig. 1:**
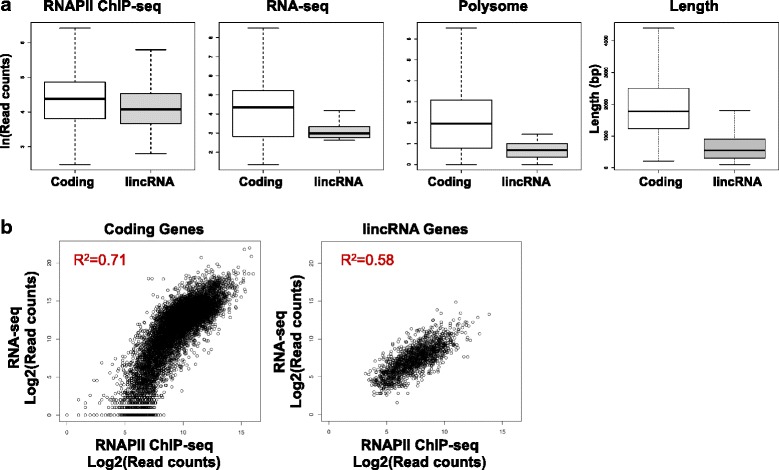
Characterization of lincRNA genes. **a** Side by side comparison of lincRNA genes (*n* = 1060) with protein-coding genes (*n* = 9730). Boxplots of RNAPII ChIP-seq (αRNAPII N-terminus), RNA-seq and polysome fractionation datasets, and size distribution of protein-coding genes (median length 1782 bp) and lincRNA genes (median length 551 bp) are shown. **b** Correlation between transcription levels (RNAPII ChIP-seq) and RNA accumulation (RNA-seq). Sequence read counts are length normalized and shown in a scatter plot. R-square values were calculated by linear model fitting

With this approach, we identified 1060 lincRNA genes (Additional file 3: Table S1) that are scattered in the genome with a median distance of 19.5 kb (Additional file 1: Figure S1c). The lincRNA genes were transcribed at a lower rate than protein-coding genes (RNAPII ChIP-seq) and the transcripts accumulated at lower levels (RNA-seq) (Fig. 1a). The lincRNA genes (median length 551 bp) were considerably shorter than the transcription units of protein-coding genes (median length 1782 bp) (Fig. 1a). The mammalian long noncoding RNA (lncRNA) catalog GENCODE v7 shows that while lncRNAs have similar exon sizes to protein-coding genes, they exhibit a bias toward two-exon transcripts, resulting in processed transcript sizes of lncRNAs shorter than mRNAs [6].

Overall, transcription (RNAPII ChIP-seq) and transcript levels (RNA-seq) correlated for coding genes and lincRNA genes (Fig. 1b). However, compared to protein-coding RNAs, lincRNA abundance was directly proportional to RNAPII occupancy, suggesting that turnover of lincRNAs may be more rapid and less variable than turnover of mRNAs.

**Fig. 2:**
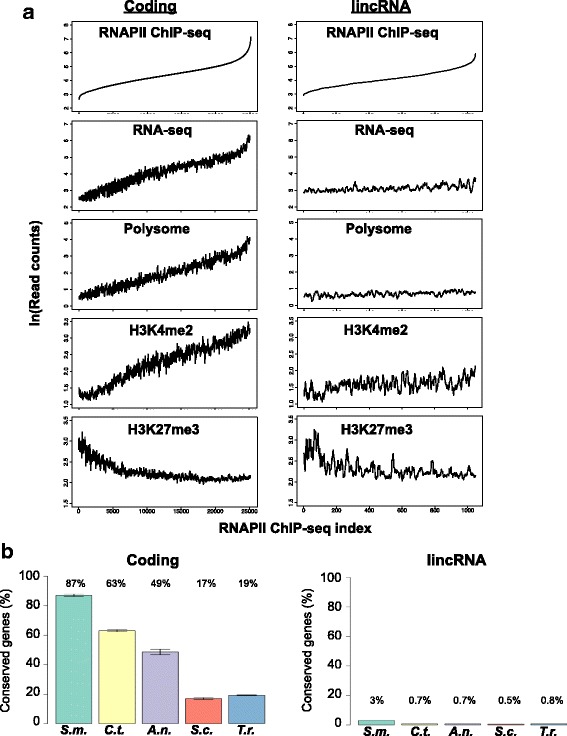
**a** RNAPII ChIP-seq index analysis. Exons of annotated protein coding genes (*n* = 25,246; left) and of the identified lincRNAs (*n* = 1068; right) were indexed according to their RNAPII ChIP-seq reads and plotted versus the read counts of RNAPII ChIP-seq, RNA-seq, polysomes RNA-seq, H3K4me2 ChIP-seq and H3K27me3 ChIP-seq, as indicated. Data are shown with a run-window averaging (see methods). Accession numbers of the H3K4me2 ChIP-seq and H3K27me3 ChIP-seq datasets are SRX550077 and SRX1818756, respectively. **b** Percentage of *Neurospora* protein-coding genes and lincRNA genes with homologies in *Sordaria macrospora (S.m.), Chaetomium thermophilum (C.t.), Aspergillus niger (A.n.), Saccharomyces cerevisiae (S.c.) and Takifugu rubripes (T.r.).* FASTA sequences of the 1478 lincRNA genes of *Neurospora crassa* were used for a discontiguous megablast (https://blast.ncbi.nlm.nih.gov) analysis. For control, 3 times 1478 protein-coding genes were randomly selected. The bars represent the percentage of input sequences that gave significant hits in each species, with + − SD for the control genes of the 3 measurement

We then ranked protein-coding genes and lincRNA genes by their corresponding RNAPII ChIP-seq coverage indices (Fig. 2a). Implementing this approach showed that increasing transcription levels of protein-coding genes correlated with the levels of total mRNA and polysome bound mRNA. The corresponding RNAPII ChIP-seq index analysis supported that lincRNAs were expressed at lower levels than the protein-coding genes and were indeed not present in polysomes. We verified that the lack of ribosome binding is not biased by the short length of lincRNAs by analyzing mRNAs that are shorter than 500 bp. These short mRNAs (*n* = 296) were significantly present in the polysome fractionation (Welch two sample t-test, *p*-value = 1.105e-07).

The presence of H3K4me2 modifications, which are associated with the transcribed regions of active genes [41], positively correlated with transcription (RNAPII ChIP-seq indices) of coding genes (Fig. 2a). However, H3K4me2 modifications were generally lower in lncRNA genes. This difference becomes in particular obvious in the fraction of highly expressed lincRNA genes, which carry considerably less H3K4me2 modifications than similarly transcribed protein-coding genes (Additional file 1: Figure S1d). In contrast, the presence of H3K27me3 modification, which is commonly associated with transcriptionally silent genes [42], negatively correlated with the transcriptional activity of both protein-coding mRNAs and lincRNAs (Fig. 2a, Additional file 1: Figure S1e).

Apart from the 1060 highly confident lincRNA genes, we detected 434 non-annotated intergenic transcripts that were, however, present above threshold in the polysome fractionation dataset. The coding potential of these transcripts was assessed by Coding Potential Calculator (CPC) [43]. Only 16 of these transcripts were predicted to have protein-coding potential (Additional file 4: Table S2). Using the BLASTX algorithm [44], the predicted protein products of only 3 of these genes were found to share homology with proteins of related species, such as *Neurospora tetrasperma* and *Sordaria macrospora* (Table 1). Taken together, our findings suggest that the list of these 16 genes may contain at least 3 novel protein-coding genes (Table 1). The remaining 418 genes with no significant coding potential were of similar length (median 451 bp) as lincRNAs and were expressed at low levels (Additional file 2: Figure S2a, b). They may therefore reflect false-positive hits of our polysome fractionation analysis (FDR = 0.05) and likely represent lincRNAs. It is also possible that some of these lincRNAs interact directly or indirectly with polysomes. Hence, these 418 intergenic transcripts with no coding potential were added to the list of 1060 lincRNAs, expanding the repertoire of *Neurospora* lincRNA genes to 1478 (see Additional file 3: Table S1).

**Table 1:** Un-annotated transcripts, which share homology with other species, with significant coding potential [43]

Coordinates (NC10)	Coordinates (NC12)	Coding Potential Score	Homology
7:1,361,800-1,362,500	7: 2,892,804-2,893,504	1.96	*N. tetrasperma, S. macrospora*
1:8,395,850-8,397,250	1:8,395,850-8,397,250	1.79	*N. tetrasperma*
6:2,116,400-2,116,700	6:2,518,550-2,518,850	0.88	*N. tetrasperma*

We then studied the conservation patterns of the 9730 *N. crassa* protein-coding genes and the 1478 lincRNA genes by using BLAST (https://blast.ncbi.nlm.nih.gov) [44] and analyzed the sequence homologies in four Ascomycota species, *Sordaria macrospora, Chaetomium thermophilum, Aspergillus niger* and *Saccharomyces cerevisiae,* and a vertebrate with a small genome, the pufferfish *Takifugu rubripes*. In stark contrast to coding sequences, *Neurospora* lincRNAs are weakly conserved. While 87% of the *Neurospora* coding genes shared homologies in the phylogenetically closest relative *Sordaria macrospora,* only 3% of the lincRNA genes were conserved between these two species and less than 1% in other species (Fig. 2b). Despite this rapid evolution, we observed strong homologies between *Neurospora crassa* and *Sordaria macrospora* for 13 lincRNAs (Additional file 3: Table S1). On close inspection, we found that 9 of these *Neurospora* lincRNAs evolved from protein-coding into noncoding gene sequences by acquiring frame disruptions. Such a mechanism is reported for the human *Xist* gene involved in X-chromosome inactivation [45, 46]. In addition, in 4 cases open reading frames were detected on the opposite strand. These RNAs may be lncRNAs that are antisense to putative coding mRNAs and their sense partners might not be expressed in our experimental setup. These instances are further discussed in the following section. Low degree sequence conservation of lincRNAs is also reported between zebrafish and human [47] and between mouse and human [48, 49]. These studies along with ours suggest that evolution of lincRNA genes is distinct from protein-coding genes and they emerge within particular lineages or species. Despite the weak sequence constraint, large numbers of lincRNAs in many species imply unconventional functional contribution by these RNAs to gene regulation.

### Identification of *Neurospora* antisense transcripts

**Fig. 3:**
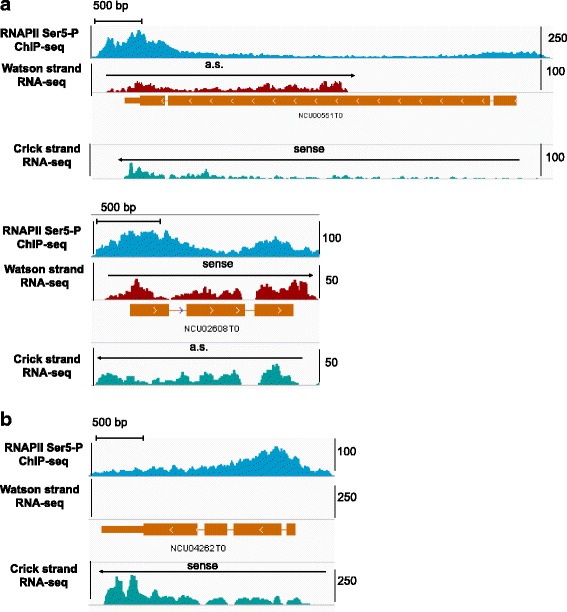
Examples of *Neurospora* antisense transcripts. **a** NCU00551, which encodes for myosin type-2 heavy chain 2, and NCU02608, which encodes for a hypothetical protein, are presented. ChIP-seq of RNAPII Ser5-P [28] and strand-specific RNA-seq datasets are shown. Direction of the transcription is depicted with an arrow for both sense and antisense (a.s.) transcripts. **b** RNAPII Ser5-P and strand-specific RNA-seq of a coding gene without an antisense transcript. NCU04262 encodes for glycosylhydrolase family 76-2 protein. The data are visualized by IGV genome browser

**Fig. 4:**
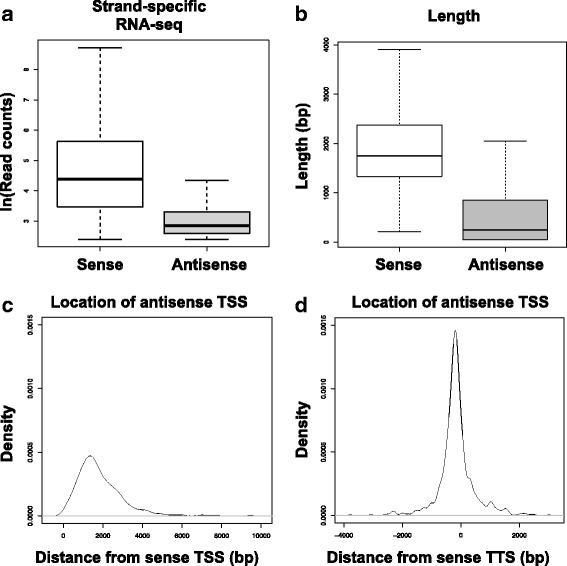
Characterization of the 826 antisense transcripts. **a** Expression levels (strand-specific RNA-seq) of antisense transcripts in comparison to their sense partners. **b** Length distribution of the antisense RNA genes (median length 251 bp) along with the corresponding sense protein-coding genes (median length 1750 bp). **c**-**d** Density diagram of antisense TSSs with respect to the corresponding sense transcription start site (TSS) or sense transcription termination site (TTS)

Natural antisense transcripts are RNA products that are made from the opposite strand of a protein-coding (sense) transcript and overlap in part or completely with their sense partner. In our analysis, antisense transcription units overlapping the same protein-coding gene were merged and scored as a single antisense transcript. We found that 826 expressed protein-coding transcripts, representing 9% of the annotated genes, have overlapping antisense partners (Additional file 5: Table S3). The identified antisense transcripts were, for highly expressed antisense RNAs, verified by the ChIP-seq profiles of Ser5 phosphorylated RNAPII (Ser5-P) [28], which is enriched at transcription start sites and the 5′ region of transcription units (Fig. 3). The expression levels of the antisense transcripts were considerably lower than of protein-coding genes (Fig. 4a) and are not correlated with the expression levels of their sense partners (Pearson’s product-moment correlation, correlation = 0.22 in dark grown samples and correlation = 0.31 in light induced samples). Moreover, the antisense RNA genes were considerably shorter (median 251 bp) than their sense partners (median 1750 bp) (Fig. 4b). We also found that antisense transcripts initiate preferentially at the 3′ ends of coding genes (Fig. 4c, d). In mammalian genomes, antisense transcription was reported to be enriched at both ends of the protein-coding genes [50, 51]. Complementarity of antisense transcripts to the 3′ ends or 3’-UTRs of sense transcripts is evolutionary more conserved and has been suggested to have regulatory roles [50, 52, 53]. The finding that *Neurospora* sense-antisense pairs overlap at the 3′ end of the coding genes suggests similar regulatory roles in this organism.

In addition, we detected 230 annotated protein-coding genes, which expressed an antisense RNA but no sense RNA under our experimental conditions (Additional file 5: Table S3). For these genes ChIP-seq profiles of Ser5 phosphorylated RNAPII (Ser5-P), which accumulates around the transcription start site (TSS), and Ser2 phosphorylated RNAPII (Ser2-P), which accumulates in the transcribed region [54] were evaluated to confirm the antisense direction of transcription (Additional file 6: Figure S3).

Previously Arthanari et al. [23] identified 540 lincRNAs and 477 antisense transcripts in *Neurospora crassa*. In our study, more than twice as many lincRNAs and antisense RNAs were identified. 62.4% of the previously published lincRNAs are detected in our dataset of lincRNAs, and 50% of the published antisense transcripts were detected in our datasets of antisense RNAs with and without an expressed sense RNA (Additional file 8: Figure S4). The larger number of lincRNAs and antisense transcripts detected in our study is due to deeper sequence coverage and to the combination of RNA-seq and RNAPII ChIP-seq analyses that enabled a better detection of genes with unstable RNA products. The lncRNAs that were not detected in our study are due to different experimental and detection pipelines.

### Light-induced expression of *Neurospora* lncRNAs

We investigated the light-dependent expression patterns of the *Neurospora* lncRNAs using the available RNA-seq datasets. We identified 687 light-inducible protein-coding genes in *wt* and 73% of the previously reported genes [30] are detected in our dataset. To detect light-induced lincRNAs, RNA-seq from this work and the published data [30] were used. We found that 181 of the identified 1478 lincRNAs are induced by light (>2× induction in either one of the time points) in both datasets (Additional file 9: Table S4). 58% (105 genes) of the light-induced lincRNAs accumulated within 30 min and the remainder of the transcripts peaked at later time points (60 min or 120 min).

Furthermore, we identified 179 light-inducible antisense transcripts (>2× induction in either one of the time points) out of the 1056 identified antisense RNAs in our analysis (Additional file 9: Table S4). 65% of these antisense RNAs were induced upon 30 min light exposure. Expression of only 35% (63 genes) of the corresponding sense transcripts was induced more than two fold at any time point, suggesting that a large fraction (65%) of light-induced antisense RNAs are regulated differently than their sense partners. In some cases light-induced antisense RNAs stem from neighboring light responsive bidirectional promoters.

### Genome-wide splicing analysis

We then performed a genome-wide splicing analysis to detect novel splice site junctions and alternative splice isoforms. 73% of the annotated introns (12,251 of 16,708) were detected with 100% overlap and 199 annotated introns were detected with mismatches (Additional file 7: Table S5). In addition, we detected 328 introns with alternative acceptor or donor sites and 249 novel introns that were previously not annotated (Additional file 7: Table S5). Together the data show that alternative splicing in *Neurospora* is rare compare to human genes, of which 40-60% has alternative isoforms [55], and only 5% of *Neurospora* protein-coding genes have the potential of being alternatively spliced.

In the majority of cases alternative splicing events produce rare transcripts (Additional file 10: Figure S5A). For instance, NCU06661 and NCU03967 encode the 60S ribosomal protein L22 and VIVID (VVD), respectively. In both cases the alternative splicing variants are rare and lead to skipping of one and two amino acid residues, respectively (Additional file 10: Figure S5B). The alternative splice variant of L22 was expressed in other species such as *Pseudogymnoascus pannorum* and *Colletotrichum gloeosporioides*. In contrast, the splice variant of VVD was not detected in the annotated proteomes of other species.

**Fig. 5:**
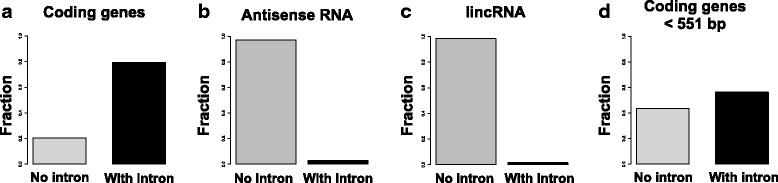
Majority of lncRNA genes do not contain introns. Fraction of (**a**) annotated *Neurospora* coding genes, (**b**) antisense RNA genes and (**c**) lincRNA genes with and without introns. **d** Fraction of intron containing genes in the subset of coding genes shorter than 551 bp (i.e. the median length of the *Neurospora* lincRNA genes)

In *Neurospora,* 80% of the protein-coding genes possess one or more introns (Fig. 5a), which have an average length of 69 bp (see Additional file 1: Figure S1a). Intriguingly, only 26 antisense RNA genes (2.5%) and 23 lincRNA genes (1.6%) were spliced (Fig. 5b, c). The absence of introns in the vast majority of lincRNAs (*n* = 1454) and antisense RNAs (*n* = 1030) is not due to their short length since protein-coding genes shorter than the median length of lincRNA genes (<551 bp) still contain introns (Fig. 5d). This finding is consistent with reports that mammalian lncRNAs exhibit inefficient splicing or remain unspliced [56, 57]. Many unspliced lncRNAs are shown to play *cis-*regulatory roles in the nucleus and are involved in genomic imprinting in mammalian genomes [58].

### *Neurospra crassa* transcriptome (NEUTRA) tool

NEUTRA tool contains a collection of multiple RNA-seq [29–31] and ChIP-seq [28–30, 59] libraries from our research group. In addition to annotated gene models, the website presents the identified lincRNAs and several statistics for gene entries, including circadian gene expression profiles and a direct comparison of gene expression levels between the wild type and several knock-out strains, namely Δ*wc1,* Δ*wc2*, Δ*sub1*, Δ*ff7* and Δ*csp1*. This tool might be useful for the scientific community.

The NEUTRA tool is publicly available at http://neutra.bzh.uni-heidelberg.de. On the **“Tools”** dialog, users can access two main sections, which are **“Search by Gene”** and **“Genome Browser”** tools.

Clicking the **“Search by Gene”** tool directs the user to a query window and a list of published [28–31, 59] and unpublished RNA-seq and ChIP-seq datasets. After selecting the datasets of interest, querying is a matter of entering a gene ID (e.g. NCU02265). The NEUTRA tool will return a table that displays detailed information about the searched gene and graphs that depict the selected RNA-seq or ChIP-seq information and expression profiles of the same gene (Additional file 11: Figure S6a).

The **“Genome Browser”** tool directs the user to a genome browser that uses the NC10 version of the *Neurospora crassa* genome model by using the open source JBrowse (http://jbrowse.org/). A gene can be searched by entering the gene ID to the search box and requested datasets can be selected from the list that appears on the left side of the browser (Additional file 11: Figure S6b).

## Conclusions

We provide a comprehensive genome-wide annotation and analysis of lincRNAs, antisense transcripts and alternative splice isoforms. We show that the prevalence of lincRNAs and antisense transcripts in *Neurospora* is higher than the previous report [23] and that 20% of the RNAPII transcripts are not coding for protein. The results provide novel insights into characteristics of *Neurospora* noncoding transcriptome. Given the large amount and diversity of these RNA species in many organisms combined with the findings that several examples of lncRNAs employ important biological functions, high-throughput identification of lncRNAs across a range of organisms is crucial to dissect their impact on gene regulation. We suggest that *Neurospora* can be a valuable model for studying functionality of non-coding RNAs.

## Additional files

Additional file 1: Figure S1. (a) Distribution of the distance between *Neurospora* protein-coding genes (median distance 1 kb). 5-percentile (134 bp) is marked. (b) The median length of *Neurospora* introns is 69 bp. The total intron length of coding genes per annotated open reading frame (ORF) is shown. (c) *Neurospora* lincRNA genes are not clustered. Distribution of the distance between lincRNA genes (median distance 19.5 kb) is shown. (d, e) (Left) Distribution of H3K4me2 and H3K27me3 enrichments in protein-coding genes (*n* = 9730) and lincRNA genes (*n* = 1060). (Right) The corresponding H3K4me2 and H3K27me3 enrichments are plotted for the upper 50% of the highly transcribed (RNAPII ChIP-seq) lincRNA genes and for the coding genes with similar transcription levels (RNAPII ChIP-seq). (PDF 139 kb)

Additional file 2: Figure S2. Characterization of the 434 non-annotated intergenic transcripts, which were detected above threshold in the polysome fractionation dataset. (a) Size distribution of protein-coding genes (n = 9730, median length 1782 bp), lincRNA genes that were below the threshold in the polysome fractionation dataset (n = 1060, median length 551 bp), lincRNA genes that were above the threshold with no significant coding potential (*n* = 418, median length 451 bp) and novel possibly coding genes (*n* = 16, median length 725 bp). (b) RNAPII ChIP-Seq index analysis of the 434 intergenic transcripts, which were detected above threshold in the polysome fractionation dataset. The data was smoothened with a window size of 100 bp. (PDF 140 kb)

Additional file 3: Table S1. List of identified *Neurospora* lincRNAs (XLSX 573 kb)

Additional file 4: Table S2. List of possibly coding genes with significant CPC scores (XLSX 28 kb)

Additional file 5: Table S3. List of identified *Neurospora* antisense transcripts (XLSX 300 kb)

Additional file 6: Table S4. List of the protein-coding genes with antisense expression only (PPTX 65 kb)

Additional file 7: Table S5. Splicing analysis: detected annotated and novel splice sites, novel alternative splice sites, splice sites detected in lincRNAs and antisense transcripts (XLSX 974 kb)

Additional file 8: Figure S3. Examples of annotated protein-coding genes with no detectable sense mRNA but only antisense RNA. NCU05980, which encodes for carboxypeptidase S1, and NCU04233, which encodes for a hypothetical protein, are shown. ChIP-Seq of RNAPII Ser5-P and Ser2-P [28], pooled RNA-Seq and strand-specific RNA-Seq datasets are presented. (PDF 42.8 kb)

Additional file 9: Figure S4. Overlap between the lists of identified lincRNAs and antisense transcripts with the previously published datasets [23]. (a) Venn diagram of lincRNA genes and possibly coding genes from this study and the published list of lincRNA genes defined by Arthanari et al. Note: numbers of genes in the diagram are slightly lower than the corresponding numbers of genes in the main text due to the computation of multiple overlaps. (b) Venn diagram of antisense RNA genes with and without expressed sense RNA and the previously published antisense RNA genes. (PDF 193 kb)

Additional file 10: Figure S5. Alternative splicing events in *Neurospora.* (a) Read count ratios of 577 annotated versus alternative spliced junctions. (b) Examples of protein versions produced by rare splicing events from NCU06661 and NCU03967, which encode for 60S ribosomal protein L22 and VIVID (VVD) proteins, respectively. Amino acids skipped in the alternative splice isoforms are highlighted in red. (PDF 43.2 kb)

Additional file 11: Figure S6. NEUTRA tool. (A) Selected statistics and expression profiles of the gene entry *frequency* (NCU02265) generated by “Search by Gene” tool. Circadian expression profile (left) and time resolved RNAPII Ser2-P ChIP-seq analysis (right) are depicted. (B) Snapshot of the genome browser. The gene model of *vivid* (NCU03967) and the selected datasets are shown. (PDF 76.8 kb)

## Abbreviations

ChIP-seq: Chromatin immunoprecipitation combined with sequencing
CPC: Coding potential calculator
lincRNA: Long intergenic noncoding RNA
lncRNA: Long noncoding RNA
RNA-seq: RNA sequencing
