# Correction to: The coding and noncoding transcriptome of *Neurospora crassa*

**DOI:** 10.1186/s12864-018-4687-9

**Published:** 2018-05-04

**Authors:** Ibrahim Avi Cemel, Nati Ha, Geza Schermann, Shusuke Yonekawa, Michael Brunner

**Affiliations:** 1 Heidelberg University Biochemistry Center, 69120 Heidelberg, Germany; 2 present address: Cellzome GmbH, 69117 Heidelberg, Germany; 3 present address: Yoshida & Co., Ltd., Tokyo, 151-8580 Japan

## Correction

After publication of the original article [1], the authors noted that Additional files 6, 8 and 9 and their legends were incorrect.

The correct Additional Files and their legends follow below.

The publisher apologises for this error.

## Additional files

Additional file 6: **Figure S3.** Examples of annotated protein-coding genes with no detectable sense mRNA but only antisense RNA. NCU05980, which encodes for carboxypeptidase S1, and NCU04233, which encodes for a hypothetical protein, are shown. ChIP-Seq of RNAPII Ser5-P and Ser2-P [28], pooled RNA-Seq and strand-specific RNA-Seq datasets are presented. (PDF 42 kb)

Additional file 8: **Figure S4.** Overlap between the lists of identified lincRNAs and antisense transcripts with the previously published datasets [23]. (a) Venn diagram of lincRNA genes and possibly coding genes from this study and the published list of lincRNA genes defined by Arthanari et al. Note: numbers of genes in the diagram are slightly lower than the corresponding numbers of genes in the main text due to the computation of multiple overlaps. (b) Venn diagram of antisense RNA genes with and without expressed sense RNA and the previously published antisense RNA genes. (PDF 193 kb)

Additional file 9: **Table S4.** Light induced RNAs in Neurospora. (XLSX 118 kb)
