# Supplementary figures and images for: The coding and noncoding transcriptome of Neurospora crassa
Source: BMC Genomics. 2017 Dec 19;18:978. doi: 10.1186/s12864-017-4360-8 (PMC5738166; doi:10.1186/s12864-017-4360-8)

Figure S1

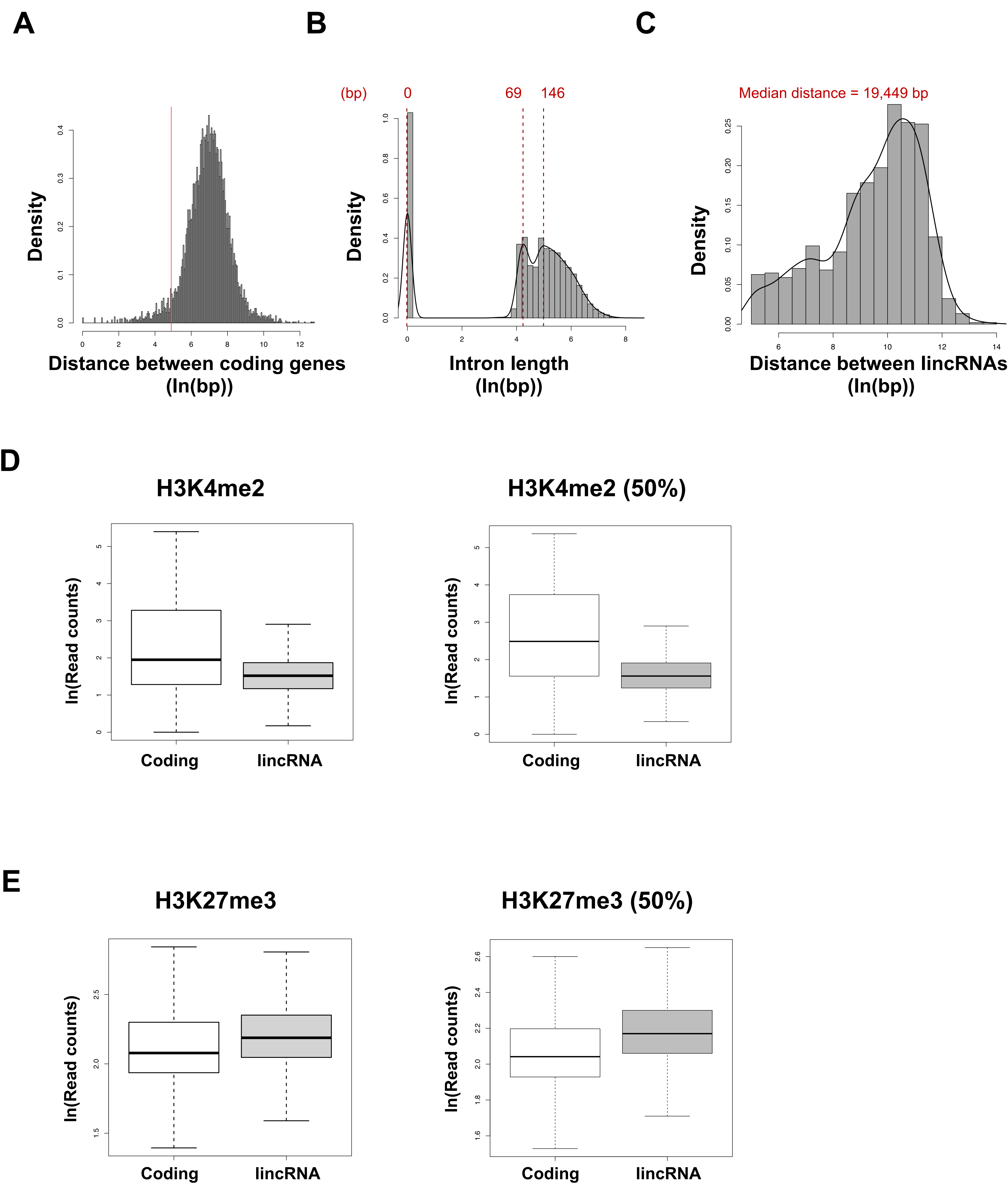

Supplement: Supplementary file 1 — (a) Distribution of the distance between Neurospora protein-coding genes (median distance 1 kb). 5-percentile (134 bp) is marked. (b) The median length of Neurospora introns is 69 bp. The total intron length of coding genes per annotated open reading frame (ORF) is shown. (c) Neurospora lincRNA genes are not clustered. Distribution of the distance between lincRNA genes (median distance 19.5 kb) is shown. (d, e) (Left) Distribution of H3K4me2 and H3K27me3 enrichments in protein-coding genes (n = 9730) and lincRNA genes (n = 1060). (Right) The corresponding H3K4me2 and H3K27me3 enrichments are plotted for the upper 50% of the highly transcribed (RNAPII ChIP-seq) lincRNA genes and for the coding genes with similar transcription levels (RNAPII ChIP-seq). (PDF 139 kb) [file 12864_2017_4360_MOESM1_ESM.pdf]

Figure S2

A

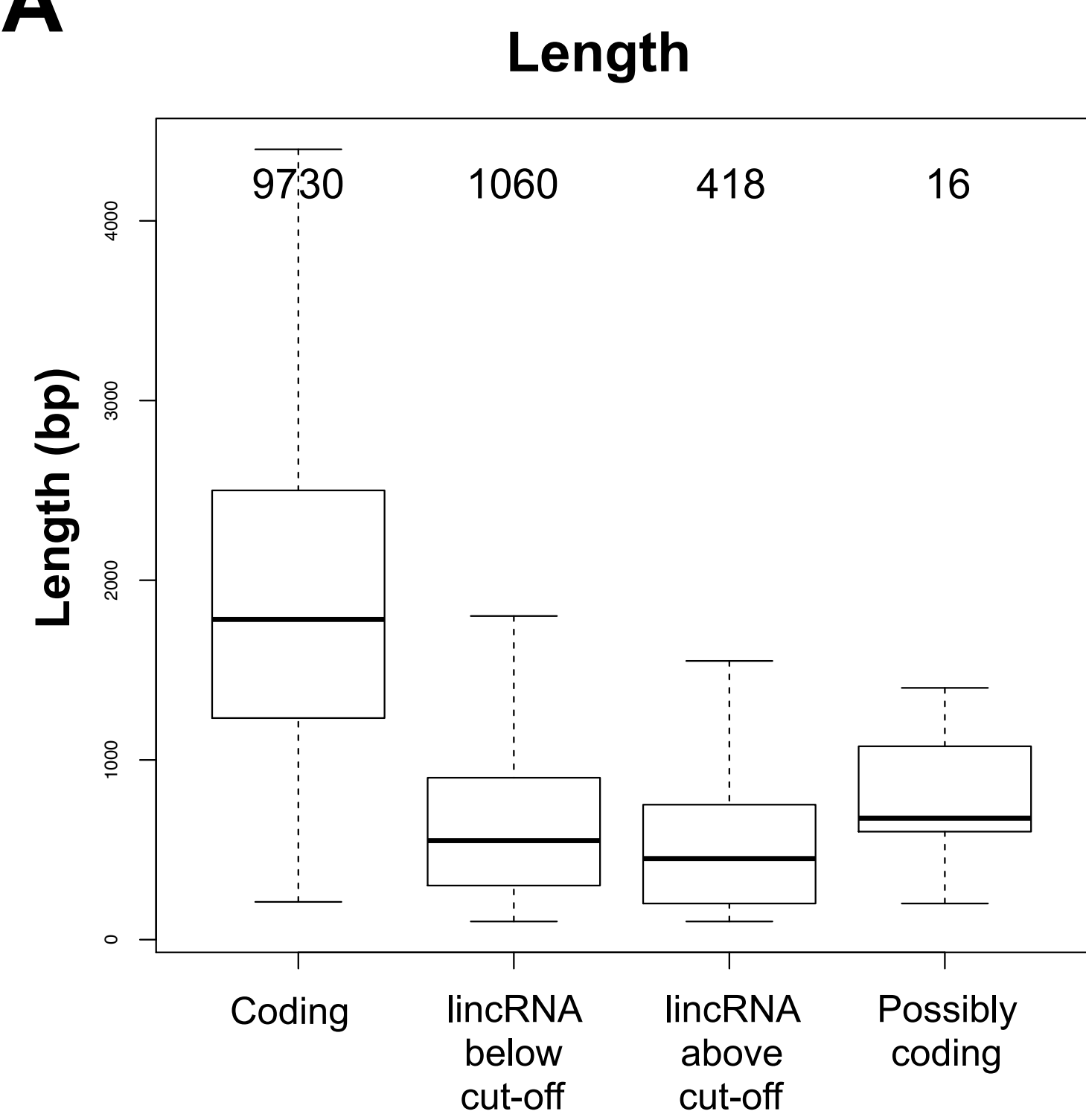

B

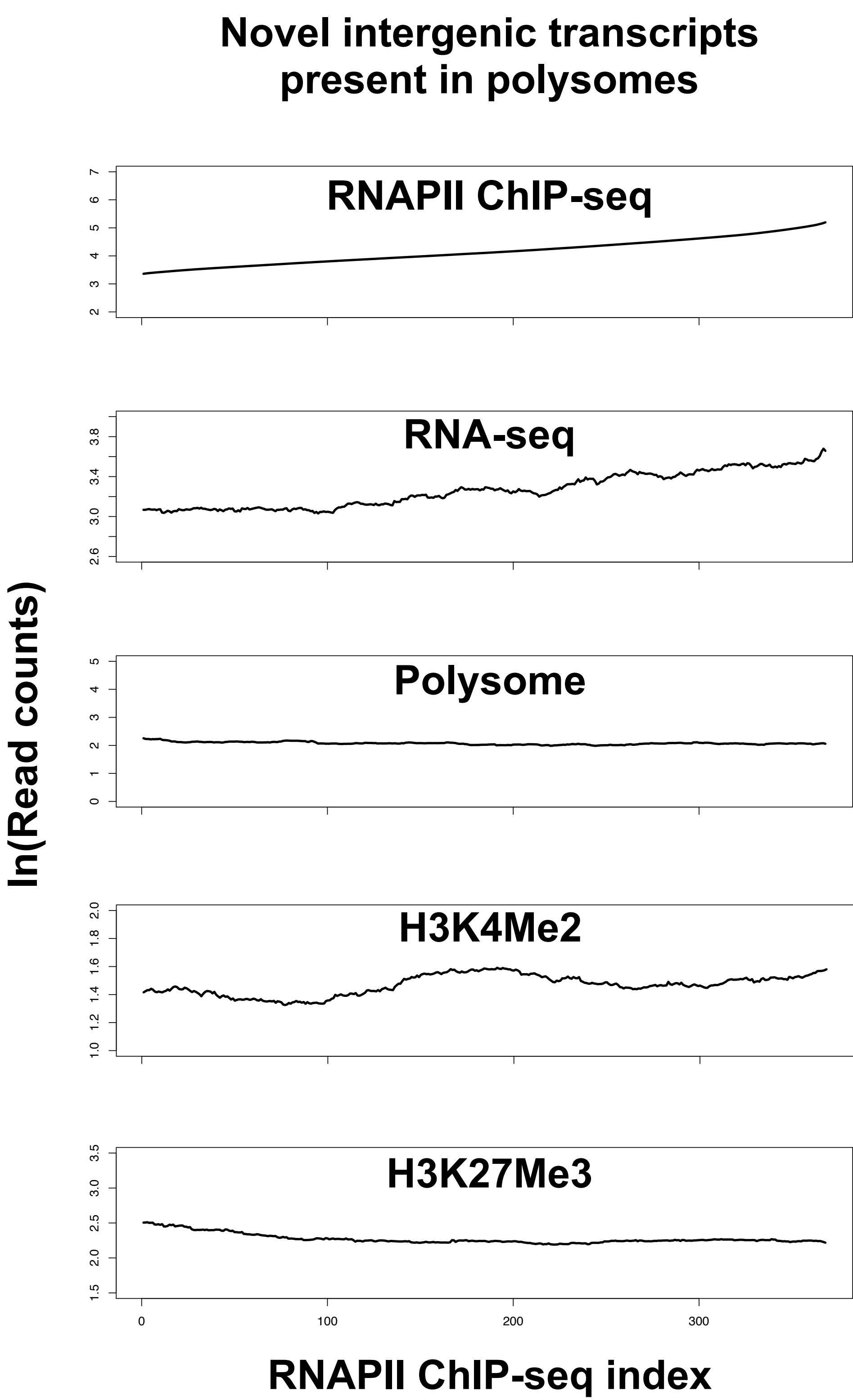

Supplement: Supplementary file 2 — Characterization of the 434 non-annotated intergenic transcripts, which were detected above threshold in the polysome fractionation dataset. (a) Size distribution of protein-coding genes (n = 9730, median length 1782 bp), lincRNA genes that were below the threshold in the polysome fractionation dataset (n = 1060, median length 551 bp), lincRNA genes that were above the threshold with no significant coding potential (n = 418, median length 451 bp) and novel possibly coding genes (n = 16, median length 725 bp). (b) RNAPII ChIP-Seq index analysis of the 434 intergenic transcripts, which were detected above threshold in the polysome fractionation dataset. The data was smoothened with a window size of 100 bp. (PDF 140 kb) [file 12864_2017_4360_MOESM2_ESM.pdf]

Figure S3

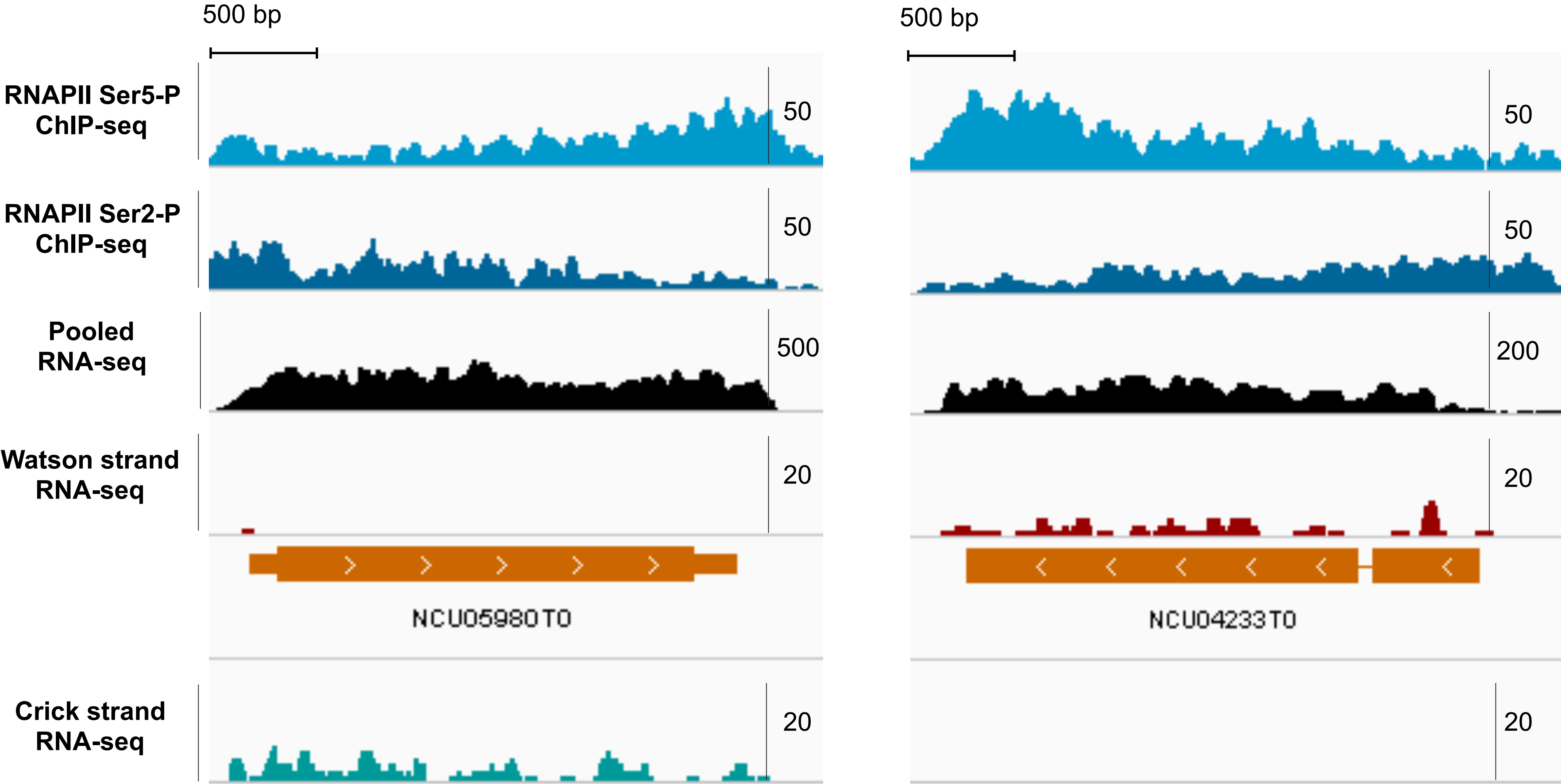

Supplement: Supplementary file 8 — Examples of annotated protein-coding genes with no detectable sense mRNA but only antisense RNA. NCU05980, which encodes for carboxypeptidase S1, and NCU04233, which encodes for a hypothetical protein, are shown. ChIP-Seq of RNAPII Ser5-P and Ser2-P [28], pooled RNA-Seq and strand-specific RNA-Seq datasets are presented. (PDF 42.8 kb) [file 12864_2017_4360_MOESM8_ESM.pdf]

# Figure S4

A

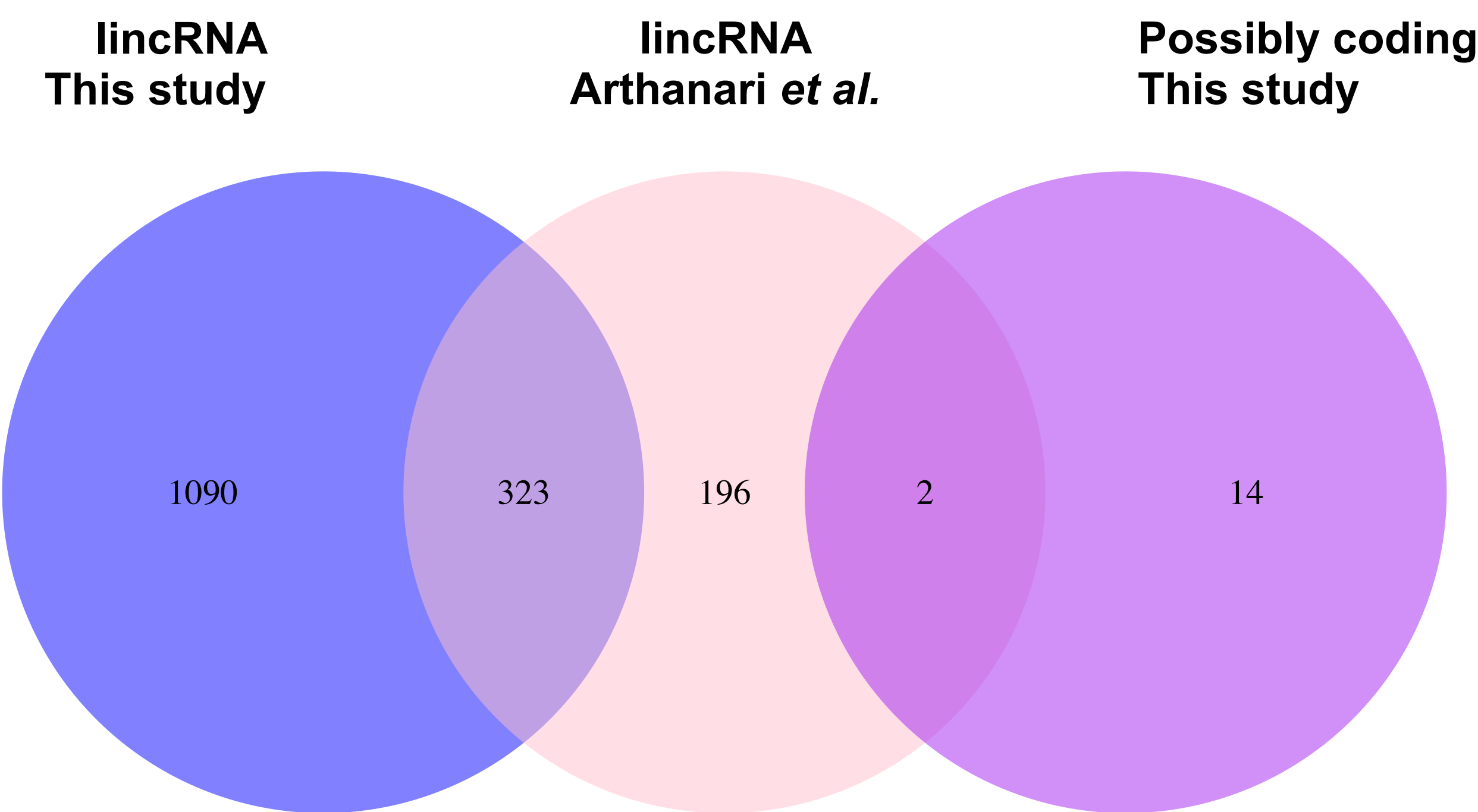

B

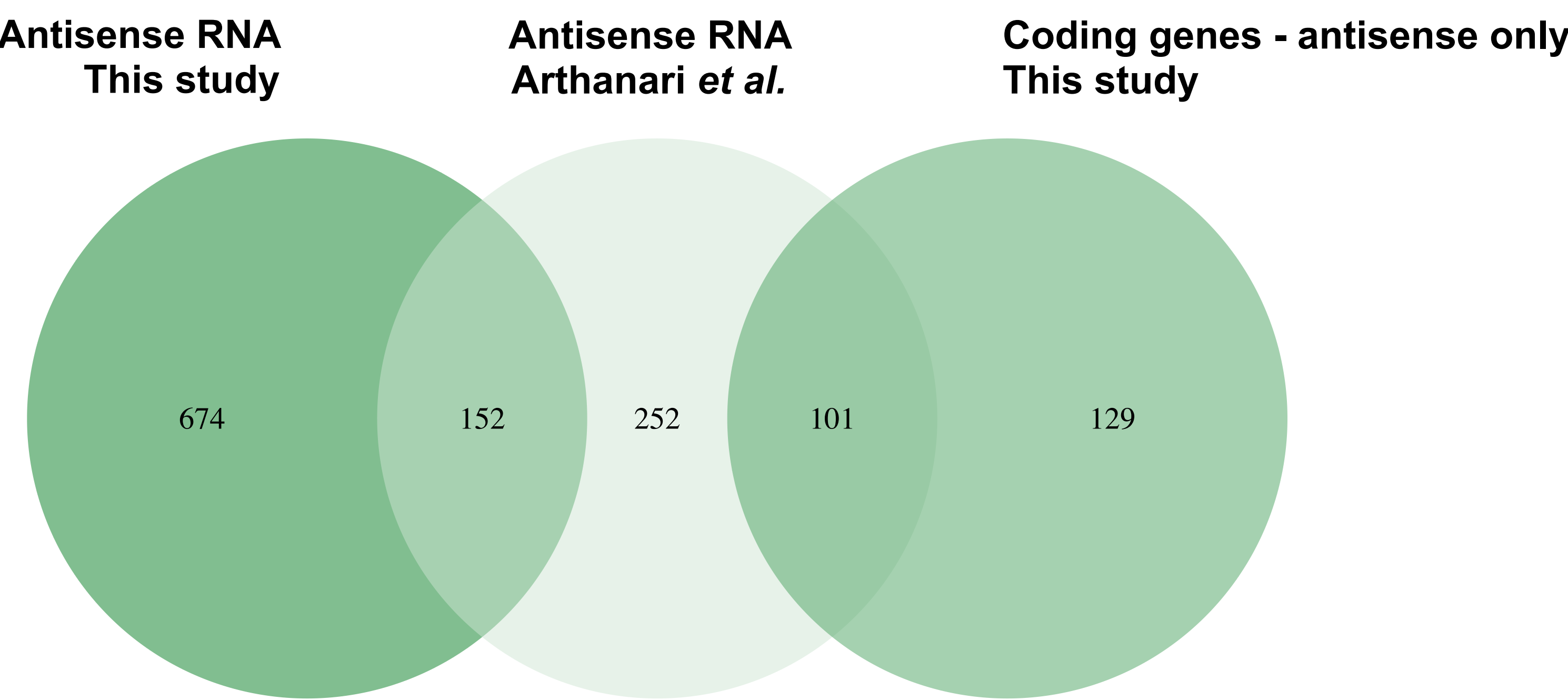

Supplement: Supplementary file 9 — Overlap between the lists of identified lincRNAs and antisense transcripts with the previously published datasets [23]. (a) Venn diagram of lincRNA genes and possibly coding genes from this study and the published list of lincRNA genes defined by Arthanari et al. Note: numbers of genes in the diagram are slightly lower than the corresponding numbers of genes in the main text due to the computation of multiple overlaps. (b) Venn diagram of antisense RNA genes with and without expressed sense RNA and the previously published antisense RNA genes. (PDF 193 kb) [file 12864_2017_4360_MOESM9_ESM.pdf]
