# Supplementary material for: The coding and noncoding transcriptome of Neurospora crassa
Source: BMC Genomics. 2017 Dec 19;18:978. doi: 10.1186/s12864-017-4360-8 (PMC5738166; doi:10.1186/s12864-017-4360-8)
Supplement: Supplementary file 6 — List of the protein-coding genes with antisense expression only (PPTX 65 kb) [file 12864_2017_4360_MOESM6_ESM.pptx]

## Slide 1
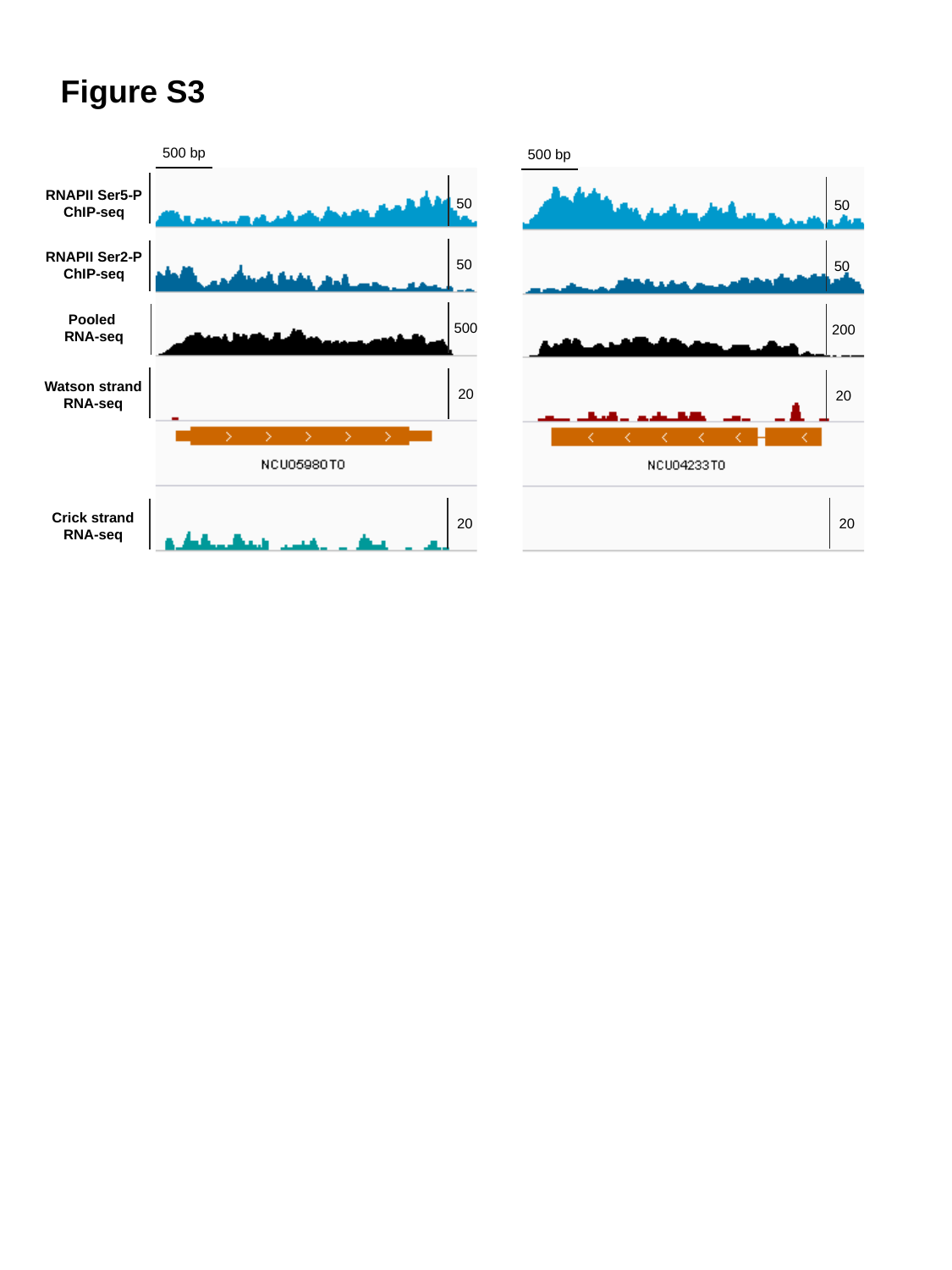

Figure S3
500 bp
50
50
500
20
500 bp
50
50
200
20
RNAPII Ser5-P
ChIP-seq
RNAPII Ser2-P
ChIP-seq
Pooled
RNA-seq
Watson strand
RNA-seq
Crick strand
RNA-seq
20
20
