# Supplementary material for: The coding and noncoding transcriptome of Neurospora crassa
Source: BMC Genomics. 2017 Dec 19;18:978. doi: 10.1186/s12864-017-4360-8 (PMC5738166; doi:10.1186/s12864-017-4360-8)
Supplement: Supplementary file 10 — Alternative splicing events in Neurospora. (a) Read count ratios of 577 annotated versus alternative spliced junctions. (b) Examples of protein versions produced by rare splicing events from NCU06661 and NCU03967, which encode for 60S ribosomal protein L22 and VIVID (VVD) proteins, respectively. Amino acids skipped in the alternative splice isoforms are highlighted in red. (PDF 43.2 kb) [file 12864_2017_4360_MOESM10_ESM.pdf]

# Figure S5

## A

In (annotated / alternative spice isoform )

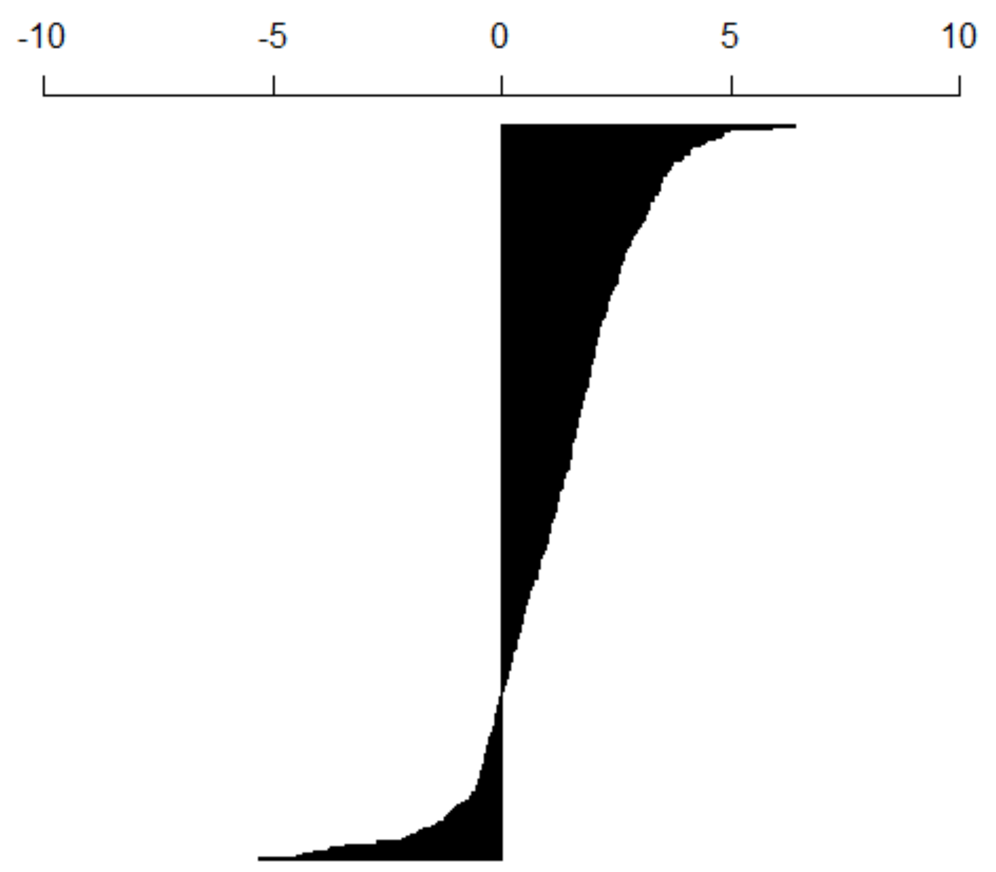


## B

**60S ribosomal protein L22 (NCU06661)**

- **Major Splicing Isoform**

MAPITKKSAKGKGPKVTKKFVINASQPASDKIFDVSAFEKFLTEKIKVEGRVGNLGDAIKISQVGDG
KIEIVAHNELSGRYLKYLTKKFLKKMQLRDWLRVVSTSRGVYELKFFNVVNDEAEEDEE

- **NCU06661 Minor Splicing Isoform**

MAPITKSAKGKGPKVTKKFVINASQPASDKIFDVSAFEKFLTEKIKVEGRVGNLGDAIKISQVGDGKI
EIVAHNELSGRYLKYLTKKFLKKMQLRDWLRVVSTSRGVYELKFFNVVNDEAEEDEE

**VIVID (NCU03967)**

- **Major Splicing Isoform**

MSHTVNSSTMNPWEVEAYQQYHYDPRTAPTANPLFFHTLYAPGGYDIMGYLIQIMNRPNPQVELG
PVDTSCALILCDLKQKDTPIVYASEAFLYMTGYSNAEVLGRNCRFLQSPDGMVKPKSTRKYVDSN
TINTMRKAIDRNAEVQVEVVNFKKNGQRFVNFLTMIPVRDETGEYRYSMGFQCETE

- **NCU03967 Minor Splicing Isoform**

MSHTVNSSTMNPWEVEAYQQYHYDPRTAPTANPLFFHTLYAPGGYDIMGYLIMNRPNPQVELGP
VDTSCALILCDLKQKDTPIVYASEAFLYMTGYSNAEVLGRNCRFLQSPDGMVKPKSTRKYVDSNTI
NTMRKAIDRNAEVQVEVVNFKKNGQRFVNFLTMIPVRDETGEYRYSMGFQCETE
