# Supplementary material for: The coding and noncoding transcriptome of Neurospora crassa
Source: BMC Genomics. 2017 Dec 19;18:978. doi: 10.1186/s12864-017-4360-8 (PMC5738166; doi:10.1186/s12864-017-4360-8)

Figure S6

A

NEUTRA (*Neurospora crassa* Transcriptome Tool)

ID	START	STOP	STRAND	NAME	CHROMOSOME	PFAM_DETAIL	NAME_SHORT	DETAIL
NCU02265	1119142	1122111	-	frequency	7			

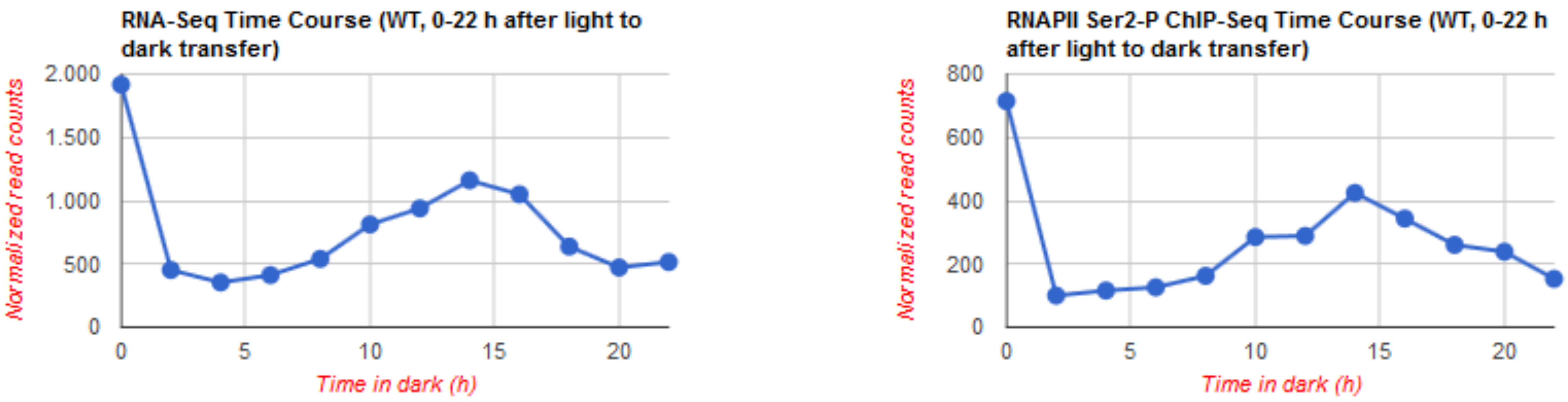

B

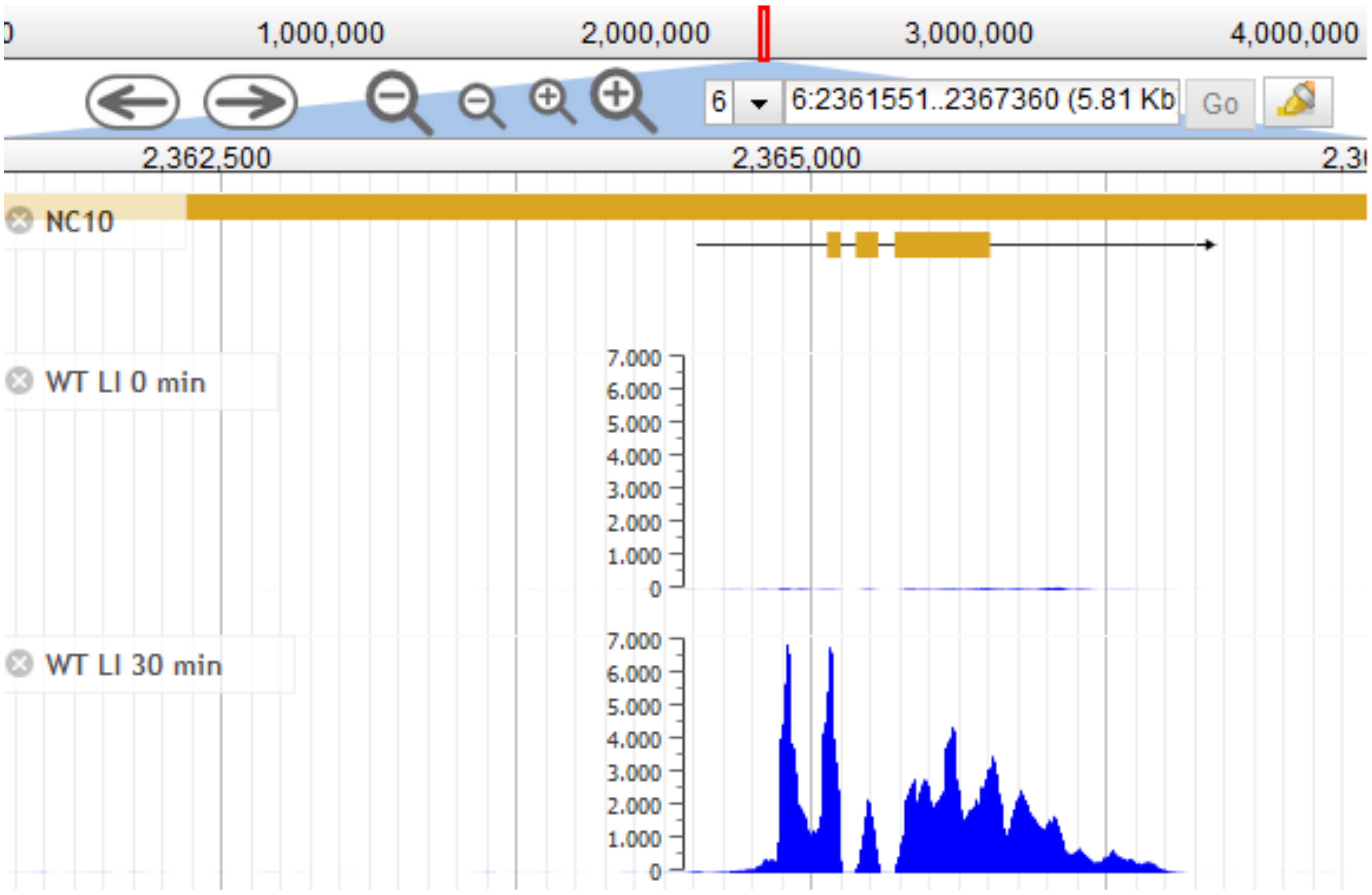

Supplement: Supplementary file 11 — NEUTRA tool. (A) Selected statistics and expression profiles of the gene entry frequency (NCU02265) generated by “Search by Gene” tool. Circadian expression profile (left) and time resolved RNAPII Ser2-P ChIP-seq analysis (right) are depicted. (B) Snapshot of the genome browser. The gene model of vivid (NCU03967) and the selected datasets are shown. (PDF 76.8 kb) [file 12864_2017_4360_MOESM11_ESM.pdf]
