# Supplementary figures and images for: Correction to: The coding and noncoding transcriptome of Neurospora crassa
Source: BMC Genomics. 2018 May 4;19:325. doi: 10.1186/s12864-018-4687-9 (PMC5935966; doi:10.1186/s12864-018-4687-9)

Figure S3

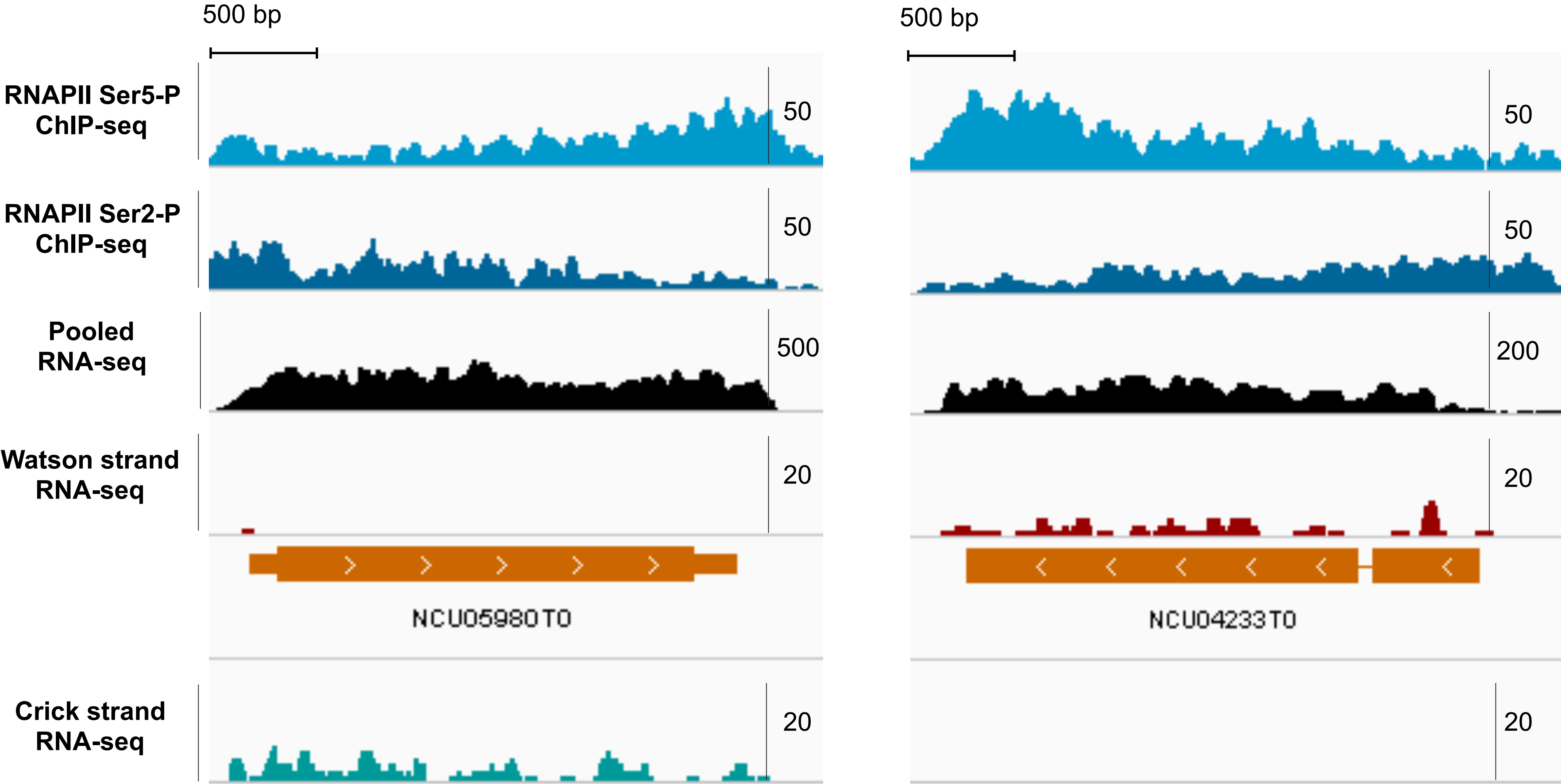

Supplement: Supplementary file 1 — Figure S3. Examples of annotated protein-coding genes with no detectable sense mRNA but only antisense RNA. NCU05980, which encodes for carboxypeptidase S1, and NCU04233, which encodes for a hypothetical protein, are shown. ChIP-Seq of RNAPII Ser5-P and Ser2-P [28], pooled RNA-Seq and strand-specific RNA-Seq datasets are presented. (PDF 42 kb) [file 12864_2018_4687_MOESM6_ESM.pdf]

# Figure S4

A

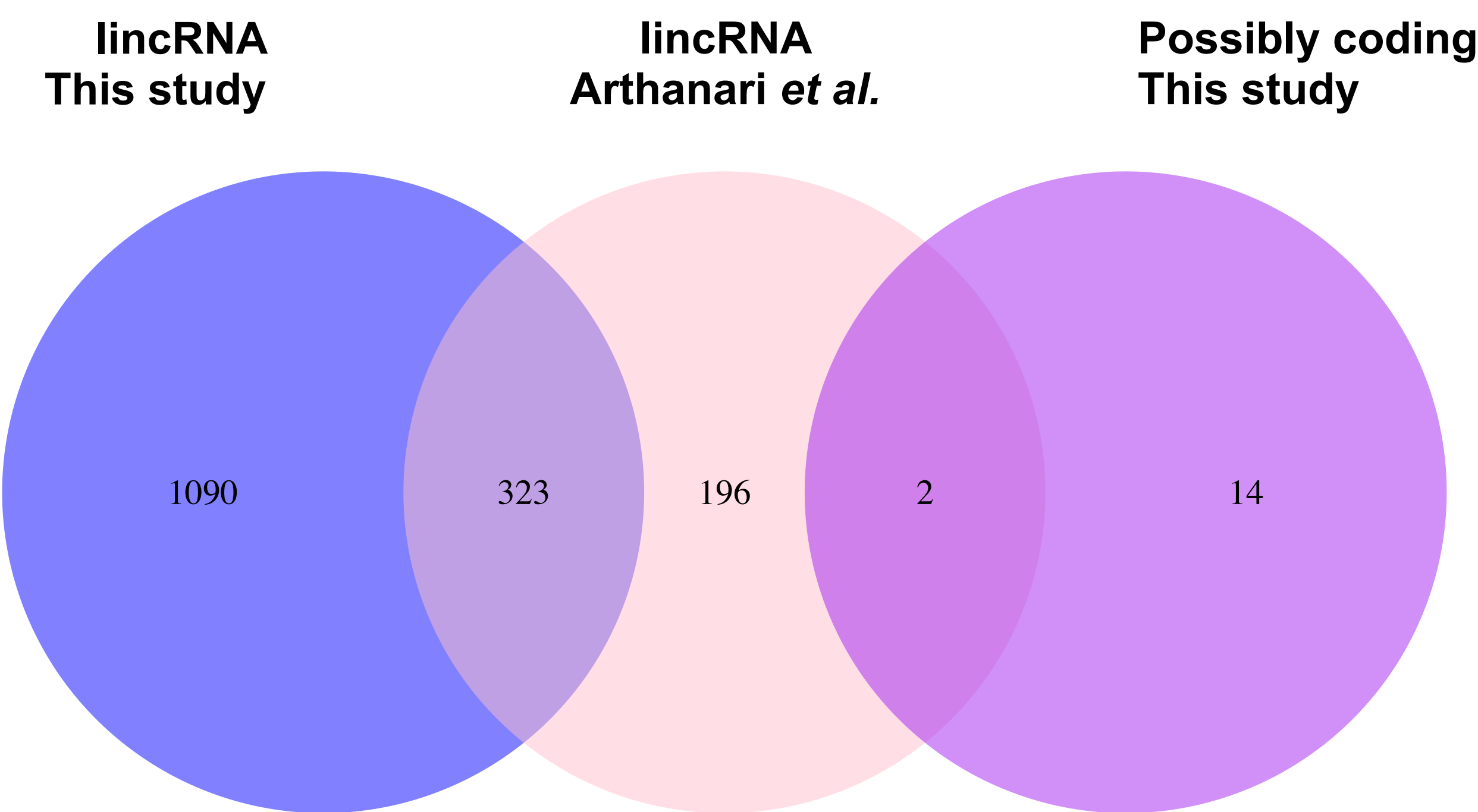

B

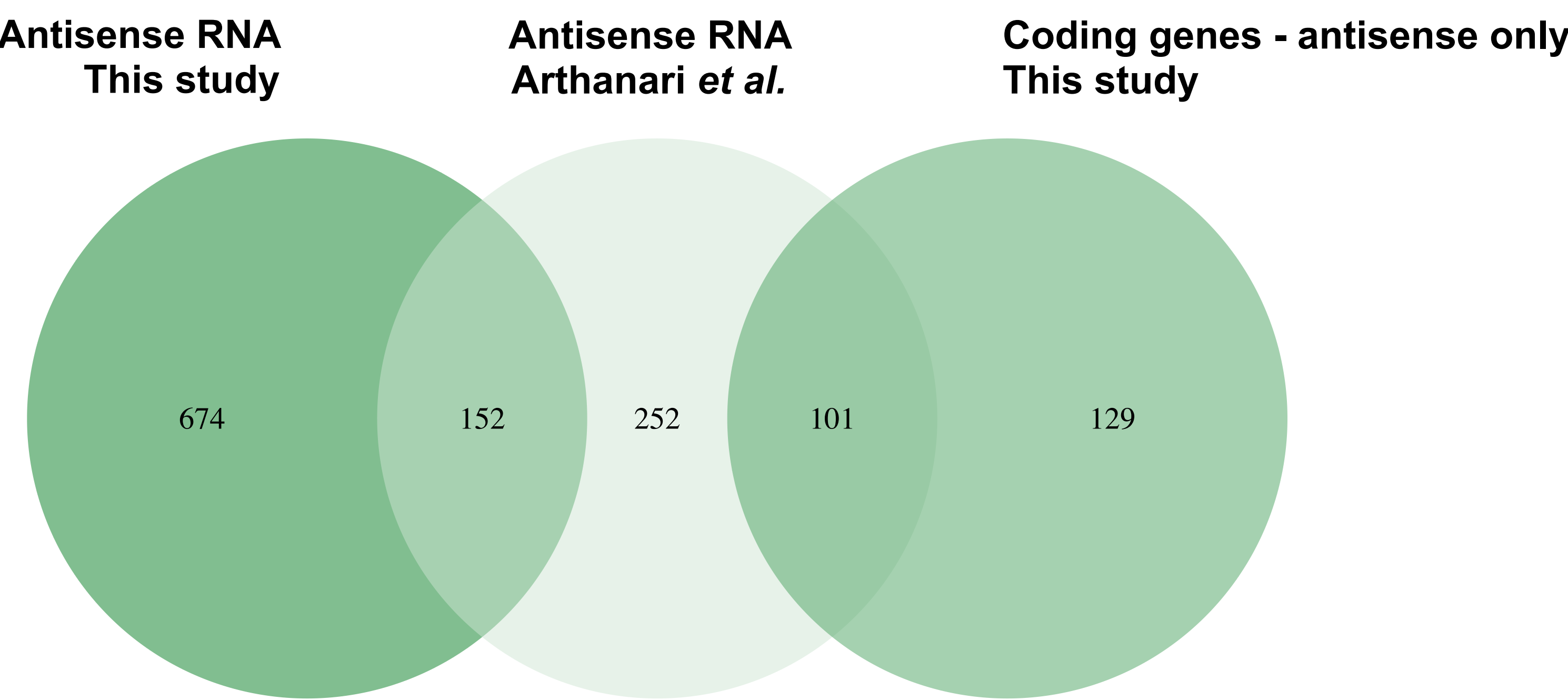

Supplement: Supplementary file 2 — Figure S4. Overlap between the lists of identified lincRNAs and antisense transcripts with the previously published datasets [23]. (a) Venn diagram of lincRNA genes and possibly coding genes from this study and the published list of lincRNA genes defined by Arthanari et al. Note: numbers of genes in the diagram are slightly lower than the corresponding numbers of genes in the main text due to the computation of multiple overlaps. (b) Venn diagram of antisense RNA genes with and without expressed sense RNA and the previously published antisense RNA genes. (PDF 193 kb) [file 12864_2018_4687_MOESM8_ESM.pdf]
